# Impact of two-level filtering on emergency medical communication center triage during the COVID-19 pandemic: an uncontrolled before-after study

**DOI:** 10.1186/s13049-020-00775-0

**Published:** 2020-08-14

**Authors:** Y. Penverne, B. Leclere, J. Labady, F. Berthier, J. Jenvrin, F. Javaudin, E. Montassier

**Affiliations:** 1 Samu 44, Department of Emergency Medicine, University Hospital of Nantes, Nantes, France; 2 Department of Medical Evaluation and Epidemiology, Nantes University Hospital, Nantes, France; 3 MiHAR lab, University of Nantes, Nantes, France

**Keywords:** Emergency medical communication center, Accessibility, System, Triage

## Abstract

**Background:**

Rapid access to emergency medical communication centers (EMCCs) is pivotal to address potentially life-threatening conditions. Maintaining public access to EMCCs without delay is crucial in case of disease outbreak despite the significant increased activity and the difficulties to mobilize extra staff resources. The aim of our study was to assess the impact of two-level filtering on EMCC performance during the COVID-19 outbreak.

**Methods:**

A before-after monocentric prospective study was conducted at the EMCC at the Nantes University Hospital. Using telephone activity data, we compared EMCC performance during 2 periods. In period one (February 27th to March 11th 2020), call takers managed calls as usual, gathering basic information from the caller and giving first aid instructions to a bystander on scene if needed. During period two (March 12th to March 25th 2020), calls were answered by a first-line call taker to identify potentially serious conditions that required immediate dispatch. When a serious condition was excluded, the call was immediately transferred to a second-line call taker who managed the call as usual so the first-line call taker could be rapidly available for other incoming calls. The primary outcome was the quality of service at 20 s (QS20), corresponding to the rate of calls answered within 20 s. We described activity and outcome measures by hourly range. We compared EMCC performance during periods one and two using an interrupted time series analysis.

**Results:**

We analyzed 45,451 incoming calls during the two study periods: 21,435 during period 1 and 24,016 during period 2. Between the two study periods, we observed a significant increase in the number of incoming calls per hour, the number of connected call takers and average call duration. A linear regression model, adjusted for these confounding variables, showed a significant increase in the QS20 slope (from − 0.4 to 1.4%, *p* = 0.01), highlighting the significant impact of two-level filtering on the quality of service.

**Conclusions:**

We found that rapid access to our EMCC was maintained during the COVID-19 pandemic via two-level filtering. This system helped reduce the time gap between call placement and first-line call-taker evaluation of a potentially life-threatening situation. We suggest implementing this system when an EMCC faces significantly increased activity with limited staff resources.

## Background

Rapid access to emergency medical communication centers (EMCCs) is pivotal to addressing potentially life-threatening conditions [1, 2]. The key role of EMCCs is well established in improving survival in the most severe patients and in allocating adequate healthcare resources. The rule that underlies EMCC activity is that the most urgent calls are immediately treated and appropriate operational resources are promptly dispatched. In France, EMCCs are organized with a first line of call takers who answer and analyze calls and then transfer calls to physicians. In emergency situations, rescue teams are immediately sent to victims*.*

Maintaining public access to EMCCs without delay is crucial in case of a disease outbreak or an exceptional health situation despite the significant increase in activity and the difficulties to mobilize extra staff resources. In this regard, the COVID-19 pandemic represents a real challenge for EMCCs. To maintain performance and quality of service [3] during the COVID-19 outbreak, EMCCs have had to change their systems. We evaluated a new two-level filtering system, as opposed to our traditional one-call-taker system, at the EMCC of the Nantes University Hospital. The aim of our study was therefore to assess the impact of a two-level filtering system on EMCC performance during the COVID-19 outbreak.

## Methods

### Description of the EMCC system

Our EMCC responds to 100% of medical and trauma calls for a population of approximately 1.4 million and received 542,413 incoming calls in 2018. Call takers have been professionally recognized since 2006 and must attend 1470 h of specific training based on a national program and completed over a 12-month period. In addition, in our EMCC, new junior call takers receive a 4-week training course after which a senior call taker accompanies them. Triage is based on the training and experience of call takers without an algorithmic system. The EMCC only receives medical or trauma calls. Other calls, including law enforcement and firefighting requests are directed to other call centers. For each call received, call takers generate a computer-aided dispatch report. Call processing consists in identifying the address or exact location of the incident, a call back number, the chief complaint, the simple symptoms and signs of emergency, the time of the occurrence and the identity and location of those involved. The call taker synthetizes and transmits the call to a dispatching physician, if available, or places the call in the virtual waiting room of the dispatching physician. Afterward, the dispatching physician performs a medical evaluation and decides on the appropriate resources to be sent to the caller. Total staffing consisted of 32 call takers, 69 emergency physicians and 44 general practioners. The number of operators differed depending on the time of day from 3 to 10 call takers and 3 to 9 physicians. Faced with a major increase in activity related to COVID 19 and the limited number of call takers, medical students were trained to answer to infectious diseases without signs of clinical severity in two days and assigned to crisis units.

### Setting and design of the study

This prospective uncontrolled monocentric before-after study was conducted at the EMCC of Nantes University Hospital from February 27th to March 25th 2020. We defined 2 periods: 1) February 27th to March 11th 2020 with the usual one-level EMCC system, and 2) from March 12th to March 25th 2020 with the implementation of a two-level filtering system.

In detail, during period one, call takers handled calls, gathered basic information (address or location of the incident, the type of emergency, obtained a call back number), gave first aid instructions if needed. Following protocols, call takers obtained information from the caller, the simple symptoms and signs that reliably indicate the presence of severe illness that would warrant urgent management, the time of the occurrence, the identity and location of those involved. Next, the dispatcher transferred the call to a dispatching physician, who performed a medical evaluation. Finally, the dispatcher sent the appropriate medical resource to the caller. During period two, between 8 AM and 10 PM, calls were answered by a first-line call taker who was asked to determine serious conditions that require immediate dispatch in a maximum of 30 s. If a serious condition was detected, the first-line call taker transferred the call to a dispatching physician, who performed a medical evaluation. If a serious condition was excluded, the call was immediately transferred to second-line call taker who gathered basic information from the caller as previously described. Thus, the first-line call taker was immediately available to handle other incoming calls. Call takers both can be first line and second line on a shift. Due to intensity of the first-line, call takers switched every 2 hours. The organization of the nightshifts (10 PM to 8 AM) did not change between period one and two, relying on a single line of call takers.

### Selection of calls

The advanced telephone system used at Nantes University Hospital’s EMCC automatically keeps track of all incoming calls. We included all of the incoming calls made between 8 AM and 10 PM from both periods. In agreement with the French national consortium [4], incoming callers who hung up in less than 10 s were considered as dialing errors and were excluded. In our EMCC, the advanced telephone system used serial call processing configured with CC Pulse + (Genesys, Alcatel Lucent).

### Outcomes

The primary outcome was the quality of service at 20 s (QS20), corresponding to the rate of calls answered within 20 s. Although other quality indicators are often used in other countries (e.g. the quality of service at 6 s, which must be ≥95% according to Dutch standards [5]), we decided to benchmark with the National Emergency Number Association (NENA) call answering standard [6] since it appears to be more adapted to the French system. QS20 represents the ability to answer incoming calls promptly and triage the request without delay. It has been proven that EMCCs save lives by promoting an appropriate prioritization and response among all incoming calls and by providing potential life-saving guidance, advices or instructions.

The secondary outcomes were descriptive statistics on the activity of the EMCC which included: number of incoming calls per hour, number of call takers per hour, call-taker occupancy rate, and average call duration. Importantly, call duration corresponded to the time before the call was transferred to the EMS physician.

### Statistical analysis

Performance indicators were extracted from our advanced telephone system and collected in a custom-designed database. Variables describing activity and performance indicators are reported by study period using mean ± standard deviation. Since the study relied on exhaustive data, significance hypothesis testing was not necessary. We nonetheless chose to use Wilcoxon tests to compare the two periods since changes could be due to random fluctuations over time.

To evaluate the change in our primary outcome (QS20), we performed an interrupted time series analysis. Indeed, the switch to a two-level filtering system on March 12, 2020 was a significant intervention that could have affected the previous trends of the performance indicators. We therefore viewed the transition to two-level filtering as an intervention that split the time series. The trend within each period is approximated by a linear regression line, defined by two parameters: an intercept (value of the time series at the beginning of the period) and a slope (average QS20 change during the period). A difference in intercept between the 2 periods indicates immediate effect of the intervention (i.e., implementation of two-level filtering), whereas a difference in slope indicates a progressive effect of the intervention. In order to independently evaluate the impact of implementation of two-level filtering, time-varying characteristics (number of incoming calls, number of connected call takers, average call duration) were also included in the model. The relationship between these variables and QS20 was tested using several types of links (linear, loglinear or exponential) and the optimal model was selected based on its likelihoods.

Since traditional linear models cannot identify inflection points on regression curves, we also used additive linear models to check the linearity of the evolution of the QS20 indicator throughout the study period. Model selection was done by cross-validation using the mgcv R package. To take confounding into account, these additive models were also adjusted to the number of incoming calls, the number of connected call takers, and average call duration.

Statistical analysis was performed using R, version 3.6.2.

## Results

### Description of EMCC activity

**Fig. 1:**
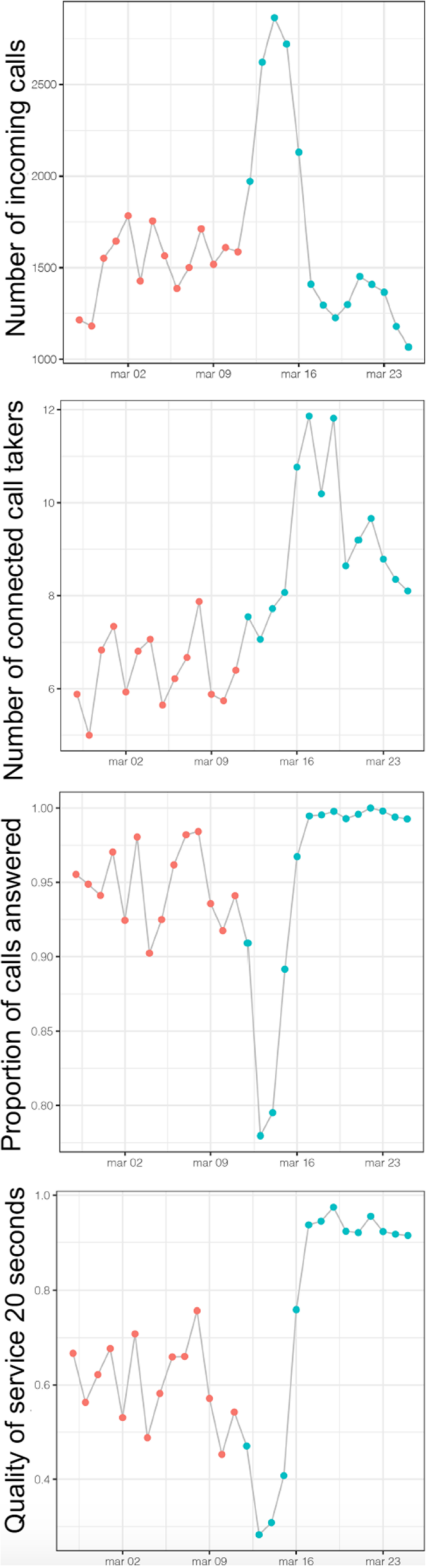
Evolution during the two study periods of different variables evaluating the activity and performance of our EDCC

Overall, we analyzed 45,451 incoming calls: 21,435 during period 1 and 24,016 during period 2. The overall descriptive and comparative statistics are available in Table 1. In period two, we observed a significant increase in the average number of incoming calls per hour (109.4 (standard deviation (SD): 24.7) vs. 122.5 (51.3), + 12.1%, *p* <  0.001), the average number of connected call takers (6.4 (1.3) vs. 9.1 (2.2), + 44.8%, *p* = 0.001), the average call duration (93.2 (14.4) vs. 104.4 (16.4), + 12.1%, *p* <  0.001) and the quality of service 20s (61% (23) vs. 76% (30), + 26,7%, *p* <  0.001) (Fig. 1). Importantly, the proportion of calls answered did not significantly differ between the two study periods (94% (1) vs. 95% (1), *p* = 0.766). Overall, the evolution of the performance indicators was similar, with a decrease at the beginning of two-level filtering followed by a significant increase.

**Table 1:** Activity and performance indicators during the two study periods in the Emergency Medical Communication Center. Overall, 392 h of activity were collected and analyzed (192 h in period 1 (February 27th to March 11th 2020, usual system) and 192 h in period 2 (from March 12th to March 25th 2020, two-level filtering)

	Overall, Mean (SD)	Period 1, mean (SD)	Period 2, mean (SD)	***p***-value
**Number of call takers per hour**	7.8 (2.3)	6.4 (1.3)	9.1 (2.2)	< 0.001
**Number of incoming calls per hour**	115.9 (40.7)	109.4 (24.7)	122.5 (51.3)	0.001
**Number of answered calls per hour**	102.7 (26.6)	98.9 (19.4)	106.5 (31.8)	0.004
**Total abandon rate (%)**	11.4 (17.3)	9.4 (9.8)	13.3 (22.3)	0.03
**Quality of service 20s**	0.7 (0.3)	0.6 (0.2)	0.8 (0.3)	< 0.001
**Call taker occupancy rate**	0.6 (0.2)	0.7 (0.1)	0.6 (0.1)	< 0.001
**Average call duration (seconds)**	98.8 (16.4)	93.2 (14.4)	104.4 (16.4)	< 0.001

### Interrupted time series analysis

**Fig. 2:**
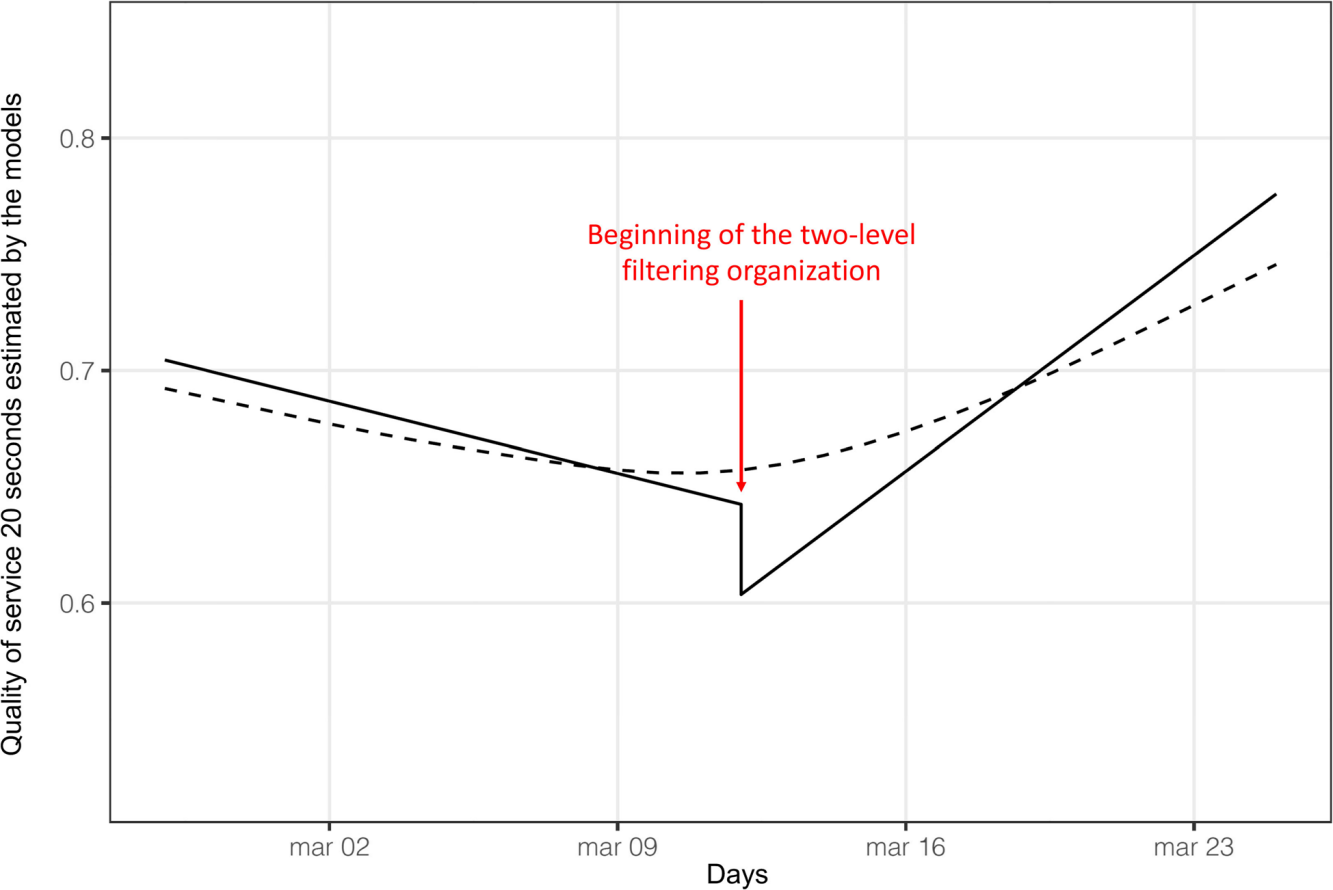
Quality of service 20 s based on the linear model (solid line) and on the additive model (dashed line), with adjustment of the average values of incoming calls, available agents and time on calls

Using an adjusted linear model, we observed a significant increase in the slope of the QS20 trend (from − 0.4 to + 1.4%, *p* = 0.01), meaning that this increase in QS20 was independent of other time-varying characteristics: number of incoming calls, the average number of connected call takers and average call duration (Table 2, Fig. 2). The additive model also confirmed the significant impact of two-level filtering. However, the intercept difference was not significant (− 6%, *p* = 0.336). No additional inflection point was apparent in the additive model (Fig. 2).

**Table 2:** Results of linear regression for the interrupted time series analysis of the quality of service 20 s. The analysis showed a significant increase in the slope after the implementation of the two-level filtering system, after adjustment in the number of incoming calls, the average number of connected call takers and average call duration

variable	estimate	95% CI	***p***-value
**Intercept period 1**	82.08	29.18 to 193.33	0.16
**Intercept difference**	−0.06	−0.17 to 193.33	0.34
**Slope period 1**	−0.01	−0.01 to 193.33	0.17
**Slope difference**	0.02	0.01 to 0.030	0.01
**Incoming calls (per 100)**	−0.03	−0.03 to − 0.02	< 0.001
**Average connected call takers**	0.09	0.07 to −0.02	< 0.001
**Average call duration (per 10s)**	−0.04	−0.07 to − 0.02	0.004

## Discussion

In the present study, we describe the activity and performance of our EMCC during the COVID-19 pandemic with implementation of a new call processing system. Indeed, given a major increase in incoming calls owing to COVID-19, we changed to a two-level filtering system with first-line call takers who were asked to determine serious conditions that required immediate action. Descriptive data showed that numerous variables affecting the quality of service changed between the two periods. We observed a significant increase in the number of incoming calls, in average call duration, and in the number of connected call takers, secondary to limited reinforcement of the workload in our EMCC. Using an adjusted linear model on these variables that changed between the 2 study periods, we observed a significant and progressive increase in the QS20 following the transition to two-level filtering. This showed that rapid access to our EMCC was maintained during the COVID-19 pandemic. This new organization helped to reduce the time gap between call placement and first-line call-taker evaluation of potentially life-threatening situations.

In France, it is possible to call the EMCC directly by a national number “15”. EMCCs, called Services d’Aide Medicale Urgente (SAMU), receive emergency medical or trauma calls. 112 is general emergency number and 20%of the calls are forwarded to the SAMU. The procedure during the first period takes several minutes during which the call taker is not available to handle incoming calls, whereas in period 2, the aim of the call duration of the first call taker’s call is 30 s maximum.

In case of an outbreak associated with an increased number of calls, it is essential to maintain rapid access to EMCCs so that emergencies can benefit from immediate dispatch of prehospital resources. During the COVID-19 pandemic, our EMCC received an increased number of calls and we tested a new system [7]. Our hypothesis was that a two-level filtering system could help to maintain EMCC performance. With a two-level filtering system, as previously described, the fist-line call takers are available to handle incoming calls and rapidly evaluate a potential life-threatening situation. Such a system could greatly improve public access to EMCCs. The French National Program of Modernization of Emergency Communications [4] promotes the interest of a two-level system during general or exceptional activity. Depending on the country, calls could be filtered before being handled by the health call taker [8]. Most French EMCCs are currently organized on a single level and can sometimes be in difficulty in case of a high number of incoming calls. Since the largest number of emergency calls concern healthcare, the French Ministry of Health has created a new health care service called “service d’accès aux soins” (SAS) [9] which includes a two-level filtering system. The aim is to improve access to the health care system and have immediate access to the expertise of a health professional in case of health emergencies. A national number (113) should be created.

The initial decrease in QS20s in the second period may be linked to the arrival of newly trained call takers with no previous experience in the field and the adaptation of new practices. In spite of this initial effect, constant improvement in QS20 is being observed and reflects better access of the public to EMCC.

### Limitations

One limitation of our study is its monocentric design which could limit the reproducibly of our findings.

## Conclusions

The principle of EMCC activity is that the most urgent calls are treated the most rapidly. Here, we found that a two-level filtering system improved the quality of service of the EMCC, independently from other time-varying variables, such as the number of available call takers, average call duration and number of incoming calls. Overall, the two-level filtering organization improved the population accessibility to the EMCC through rapid triage and the identification of life-threatening emergencies. Since EMCCs are designed to dispatch appropriate and rapid resources for potentially out-of-hospital life-threatening conditions, a two-level filtering system is a very adequate solution in a crisis situation with a massive increase in incoming calls. This system should be widely implemented in case of a disease outbreak such as the COVID-19 pandemic or other situations that lead to a massive increase in EMCC activity with temporarily limited staff resources and/or difficulties in answering incoming calls.

## Data Availability

Please contact the authors for data requests.

## Abbreviations

EMCC: Emergency medical communication center
QS20: Quality of service at 20 s
SAMU: Service d’aide médicale urgente
SAS: Service d’accès aux soins
